# Girl–boy differences in perceptions of health determinants and cancer: a more systemic view of girls as young as 6 years

**DOI:** 10.3389/fpubh.2023.1296949

**Published:** 2023-12-19

**Authors:** Chloé Gay, Maéliane Deyra, Pauline Berland, Laurent Gerbaud, Frank Pizon

**Affiliations:** ^1^Université Clermont Auvergne, Clermont Auvergne INP, CNRS, Institut Pascal, Clermont-Ferrand, France; ^2^Université Clermont Auvergne, Clermont Auvergne INP, CHU Clermont-Ferrand, CNRS, Institut Pascal, Clermont-Ferrand, France

**Keywords:** girls, boys, children, gender, health conceptions, health determinants, cancer determinants, perceptions

## Abstract

**Introduction:**

To model and analyze the differences between girls' and boys' conceptions of the determinants of health and cancer, as expressed and perceived by children and adolescents.

**Method:**

A multicentric qualitative study was conducted in five schools (ages 6–11 years), four middle schools (ages 11–15 years), and three high schools (ages 15–18 years). A multi-phase protocol (phase 1 uses the e.Photoexpression© and phase 2 uses the Photonarration) enables children and teenagers to express themselves through photography and storytelling.

**Results:**

A total of 4,174 qualitative productions were produced by 1,068 children, of which 47% were girls and 53% were boys, all in the ages of 6–18 years. From the results of the productions, it can be noticed that children mentioned and were aware of 30 determinants of health and cancer. The three determinants most mentioned were “Consumption of psychotropic drugs”, “Diet”, and “Harmful environment”. Among these 30 determinants, some were mentioned to a greater or lesser extent by girls and boys. These significant gender differences are present for 20 determinants of health and cancer. These differences evolve with age: (1) In elementary school (ages 6–11), girls gave significantly more importance (*p* < 0.05) to 11 determinants, while boys attached significantly more importance (*p* < 0.05) to 2 determinants. (2) In middle school (ages 11–16), girls gave significantly (*p* < 0.05) more importance to 12 determinants, while boys gave significantly (*p* < 0.05) more importance to one determinant. (3) In high school (ages 15–18 years), girls gave significantly (*p* < 0.05) more importance to 13 determinants. There was no significant difference (*p* < 0.05) in favor of boys for high school students. Girls also have a more systemic view of health determinants than boys. The increase in the number of determinants cited by girls is significant (*p* = 0.017) between the ages of 6–11 and 15–18 years. This gap widens with age (+1.45 determinants) for girls and (+0.68 determinants) for boys between elementary school and high school.

**Conclusion:**

The determinants identified as predominantly female or male, as well as age-related specificities, constitute a resource for effective preventive action, as close as possible to the needs and particularities of a population. This mapping of people's conceptions could provide a decision-making aid in defining the strategic orientations of prevention policies.

## 1 Introduction

In current times, to promote health and reduce the risk of developing certain diseases, including cancer, it is internationally recognized that it is necessary to act on the determinants of health, which are “the personal, social, economic and environmental factors that determine the state of health of individuals or populations” (1). The combination of their effects creates living conditions that influence overall health in specific ways (2). The influence of all these determinants on health has been widely demonstrated (3), and the current question is to understand how they work and how we can intervene as effectively as possible in prevention (4).

The major challenge of this research is to document the literature on the conceptions of the determinants of health and cancer from the point of view of children and adolescents so that prevention policies aimed at them best respond to their concerns. Indeed, a review of the literature (5) shows a small number of qualitative data available with the opinions of children and adolescents on the determinants of health. Too little voice is given to children and adolescents regarding their health and what determines it. This research allowed this population to express themselves on their conceptions of what determines health and cancer. This publication aims to relay their words and opinions so that their perceptions of health and these determinants are taken into account in public policies and thus meet their real needs.

Prevention interventions that are pre-established within a precise theme, without taking into account the public's conceptions, cannot be effective because they are too far removed from the conceptions of individuals. It seems essential to identify what can be built on to get closer to the zone of proximal development and take effective action (6). To understand these perceptions, Pizon et al. demonstrated the importance of investigating health conceptions (7, 8). However, internationally, there is little research available on how children and adolescents perceive the determinants of health and cancer (5).

The term “conception of health” encompasses what enables individuals to characterize their health and what determines it from a biopsychosocial perspective (8).

Our team has developed methodologies for collecting and analyzing mass qualitative data, enabling us to measure how a cohort identifies and prioritizes preventive interventions (9). To survey a large population, the use of images proves highly relevant. These approaches value the voice of children and adolescents, demonstrate their ability to express themselves on complex subjects, and underline the importance of taking into account their often underestimated conceptions to adapt prevention strategies (8, 10). These methodologies, which form the basis of our study, combine the use of multi-phase data collection using photography and images, e.Photoexpression© and Photonaration.

Combined with a mixed analysis of data and their pictorial representation in the form of qualitative mass data modeling, these data collection tools are deployed specifically for children and teenagers (9, 11). Our study makes it possible to identify global and collective trends within a group as well as the most fragile individual profiles or those who are more at ease. These results at different scales provide decision support at several levels for the implementation of decisive actions on the health of populations. At the level of the individual's living environment, a better understanding of the population in a given area can help in defining the strategic orientations of public health policies. At the level of the individual (age and gender), those working in the field of prevention will be able to identify the specific characteristics of their audience, with the aim of targeting and adapting their interventions as closely as possible to their needs to enhance their effectiveness.

The aim of this research is to model and analyze the differences in conceptions of the determinants of health and cancer between girls and boys, as expressed and perceived by schoolchildren and adolescents aged between 6 and 18 years. This study will also validate our diagnostic and decision-making methodology, enabling us to prioritize preventive actions by demonstrating that it is necessary to take design into account before any intervention.

## 2 Materials and methods

### 2.1 Study framework

This is a qualitative human and social sciences (SHS) study of schoolchildren and teenagers. The study was conducted in 12 schools in the Auvergne-Rhône-Alpes region (AURA, France). The schools were selected on a voluntary basis and according to a panel representative of the general population. The schools, middle schools and high schools, that participated in this study are establishments located in priority or non-priority education zones, those located in rural and urban geographic areas, and those offering general and technological education courses. The recruitment period took place from January 2019 to March 2021. A total of 1068 children and adolescents in the ages of 6–18 years took part in this collection. All children willing to participate were included in this study. Adult dictation was used for children who had not yet mastered writing. Our methodologies based on the use of images are non-invasive and allow everyone to express themselves. Among all these children, three had a painful experience linked to cancer (loss of a parent). These families, therefore, benefited from an interview with the teacher beforehand to answer any questions they might have and to obviate any anxiety they might feel about the study. The children were given the option of leaving the session at any time if they so wished; however, none of the children made use of this option.

### 2.2 Data collection

Data collection was carried out according to the Determ'ados methodology published in 2021 (9): “*Joint Use of e.Photoexpression*© *and Photonarration: What Methodological Added Value?”* e.Photoexpression© and Photonarration are complementary and inseparable to bring out ideas about health and cancer. The Determ'Ados survey methodology, in two successive phases (e.Photoexpression© and Photonarration), therefore, allowed us to reveal that children, from a very young age, have a wide range of conceptions of the determinants of health. The range of designs indicates the effectiveness of the method. By adapting to the level of children's writing (dictated to adults), these collection tools allow a heterogeneous panel of children (age and social context) to express themselves freely, to verbalize and mobilize conceptions that may or may not be revealed, and to express in a more or less distinct manner. They have their own conceptions and manage to structure their thinking in an explanatory logic that it is essential to take into account. These different approaches offer new content and are, above all, complementary. They create a funnel effect, moving from the global to the specific determinants, thus forming a reassuring cognitive journey for the child. A general entry on health then oriented toward cancer favors a progressive approach. The added value of the multi-phase lies in this approach, a progressive methodological operation that facilitates the support of the child in a mental process of adaptation to the subject.

#### 2.2.1 Phase 1 “e.Photoexpression©”

This is an image-based mediation tool e.Photoexpression (8) consisting of a corpus of 40 copyrighted photographs. This corpus of 40 photographs was designed to encourage the emergence of health-related conceptions. To this end, each of the photographs meets esthetic, significance, and heterogeneity criteria. These criteria enable the photos to be suggestive and meaningful to people while at the same time being open to a variety of readings. The broad spectrum of these photos allows each participant to find the one(s) that allow(s) them to express themselves. Based on the three criteria mentioned above, photography enables us to co-construct a reality (or the object of research) in this triadic interaction among researcher–photographer–subject. The photograph “made available” by the researcher is thus reappropriated by the subject, who offers a personal interpretation. It is important to point out that these photos are in color to reflect the current “media” dimension (image society, media, etc.).

In this study, this tool was used to identify the determinants perceived as exerting a favorable or unfavorable influence on one's health. The children and teenagers chose two images from 40, which best responded to the proposition: “Choose an image which, in your opinion, represents good health” and “Choose an image which, in your opinion, represents bad health”.

Each child wrote down the reasons for their choice of images.

#### 2.2.2 Phase 2 “Photonarration”

Photonarration (8) consists of bringing out design systems by cutting, assembling, and collaging images from magazines. The eligibility criterion for magazines offered to children and teenagers is a wide thematic diversity (sports, nature, hobbies, health, food, leisure, medicines, hygiene products, and other consumer products) targeting different ages (children, adults, and seniors).

In this study, Photonarration was used to identify the determinants perceived as influencing the occurrence or non-occurrence of cancer, as well as children's systemic perceptions of them. Participants cut out, glued, and assembled the desired images on an A3 sheet in response to the proposition: “Glue here the images you've selected that represent what can lead to cancer” and “Glue here the images you've selected that represent what makes it possible to avoid getting cancer”. Each production (A3) was associated with a text describing the reasons for choosing the images.

### 2.3 Data analysis

#### 2.3.1 Qualitative analysis

The data analyzed are the verbatims written by children and teenagers during the e.Photoexpression© (phase 1) and Photonarration (phase 2) sessions, entered in full in an Excel spreadsheet. Each reference to a health determinant mentioned in the children's verbatims was reported and classified in the Excel spreadsheet. This classification of the verbatims was represented by strata at two different levels, depending on the degree of granularity of the pupils' comments: domains and categories. To preserve the authenticity, finesse, and detail of the elements addressed by the children and teenagers, a large number of domains and categories were created. This classification works both upward and downward, always with a view to keeping as close as possible to what the children have to say. Exchanges between researchers from different disciplines have greatly enriched the analysis and the way of looking at the data. Once data saturation had been achieved, convergent points were grouped by a block of meaning (12): the larger the block of meaning, the closer we came to the domain; the smaller the block of meaning, the closer we came to the category. We constantly went back and forth, providing feedback at all levels: between the source data, the conceptual and theoretical model of health determinants (9), and exchanges with the multidisciplinary research team. All this to-ing and fro-ing enabled us to maintain the authenticity and accuracy of the data and the granularity of the source data to avoid over-interpretation. We thus adjusted and stabilized the modeling of health and cancer determinants, which were divided into 29 categories and seven domains (11).

The domains represent individual or environmental/collective health determinants. Each category is correlated with the domain to which it belongs, providing additional information. The categories represent favorable or unfavorable determinants of health. The domains represent a dominant idea that groups and structures the categories. A category groups together elements of vocabulary or information organized according to a certain number of common semantic or grammatical criteria.

This study requires simultaneous data collection and qualitative analysis. Indeed, it is the analysis that allowed us to complete the recruitment of the schools when the data saturation point was reached. In fact, it was the qualitative analysis that, once the data had been saturated and the seven domains and 29 categories were stabilized, enabled us to complete the recruitment of schools.

In preparation for the statistical analysis, all verbatims were indexed to the model of seven domains and 29 categories created during the qualitative analysis. The exchange between qualitative and quantitative data avoids over-interpretation and helps guarantee the validity of the results. All data were collected by the same interviewers. Double coding was used for qualitative data analysis and indexing. In the event of a dispute, a third party was called in. These measures also served to limit bias.

#### 2.3.2 Statistical analysis

A descriptive quantitative analysis was carried out. Domains and categories were described in terms of numbers and percentages.

Statistical analyses were performed with SAS v9.4, and a *p*-value of <0.05 was considered statistically significant. SAS is database management and statistical analysis software. The source of SAS v9.4 was SAS Institute Inc., Cary, NC, USA and SAS (r) Proprietary Software 9.4 (TS1M3), licensed to LICENSE MESR SAS RECHERCHE PCW, Site 70109802.

For the average comparison analysis of different groups (trends in the number of health and cancer determinants cited by girls and boys aged between 6 and 18 years), we used ANOVA as a statistical formula. Percentage comparisons were made using the chi-square test (pictorial representation of significant differences in conceptions of health determinants and cancers between girls and boys in the ages of 6–11, 11–15, 15–18, and 6–18 years).

### 2.4 Ethical considerations

This study was conducted in accordance with the “consolidated criteria for reporting qualitative research (COREQ)” recommendations. This research was ethically certified by the Comité de Protection de Personne sud EST VI (France). Teachers in the Auvergne-Rhône Alpes education authority were recruited on a voluntary basis. All children and parents who gave their consent participated in the study.

The methodology used enabled us to approach the subject of cancer from the children's point of view in a benevolent and ethical manner. To reassure adults (families and teachers) who might find the subject complex and unsuitable for children, andragogical information and presentation of the project were carried out in the field. These interventions were meaningful for the children who showed a real enthusiasm for participating in this research. The choice of tools was explained to the children. In fact, image-based mediation tools allowed us to remain off-center, avoiding talking about ourselves but remaining focused on the theme at hand. The child who “leads” their discourse based on the photographs then becomes an actor in the collection, providing their own interpretation of the photographs they have chosen.

Moreover, the approach based on the theme of global health and the limited choice of images (good health and bad health) guarantees a step-by-step progression that respects the child's cognitive process of adaptation and acceptance of reflection. Choosing two photographs is a pleasant and motivating activity, quick to implement, and enjoyable. Children tend to be very interested in any form of pictorial representation, especially photographs. Photographs provide a clear picture of the point of view expressed, which can then be explored with them in greater depth, and reinforce the building of a relationship of trust between researchers and participants.

## 3 Results

### 3.1 Descriptive data

**Figure 1:**
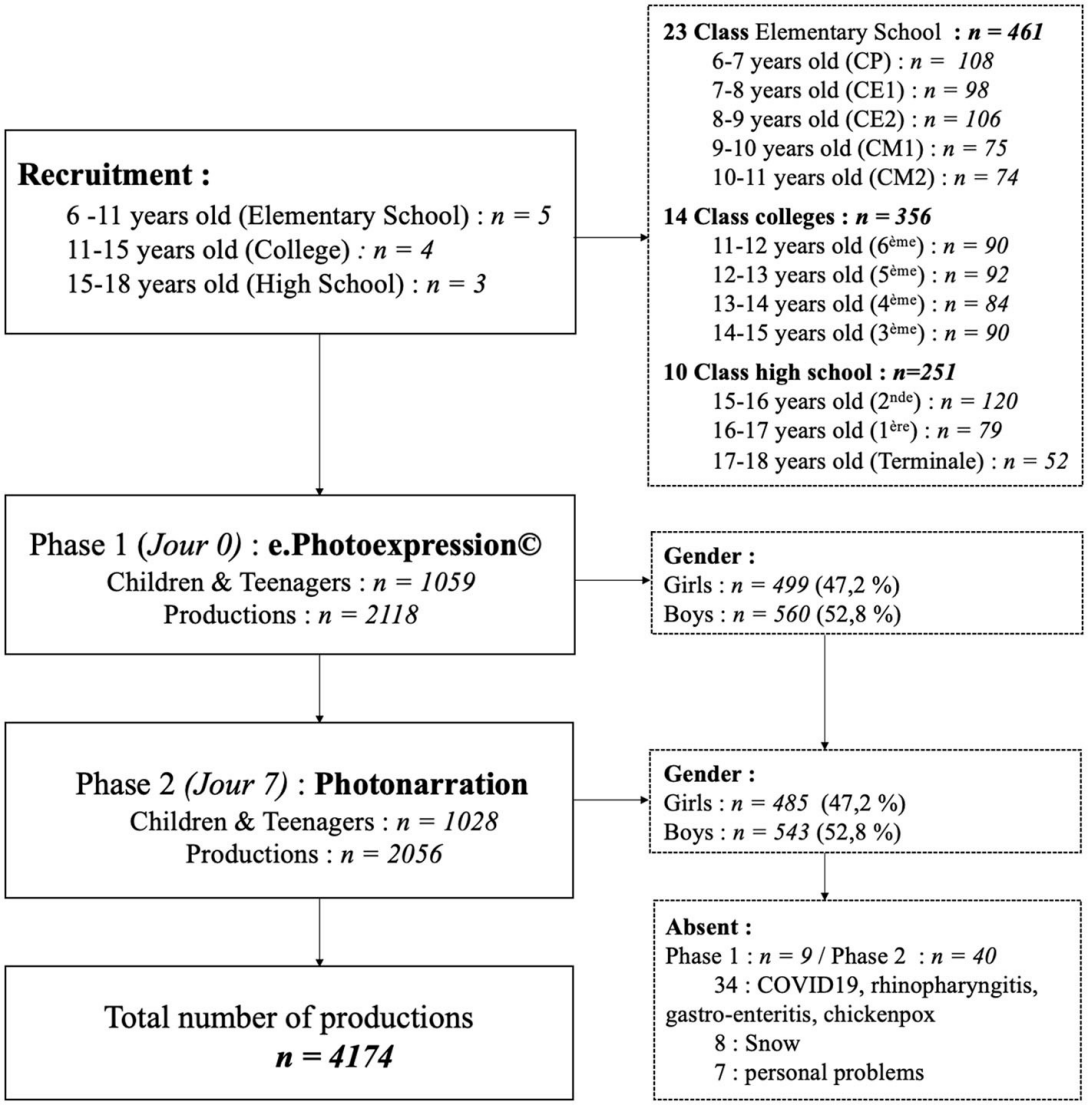
(Flow diagram) illustrates the descriptive data from this study.

Data were collected from five elementary schools, four middle schools, and three high schools in the Auvergne-Rhône-Alpes (AURA) region of France (Figure 1).

During phase 1 of the survey, 1,059 children and teenagers took part and produced 2,118 productions. In phase 2, 1,028 children and teenagers were interviewed and 2,056 productions were collected.

In all, 4,174 productions were produced (four productions per child) by 1,068 children.

The reasons given by the nine pupils absent during session 1 and the 40 absent during session 2 were snow for 8 of them, illnesses for 34 of them [COVID-19 (13), rhinopharyngitis (5), gastro-enteritis (7), chickenpox (2)], and personal problems for seven of them.

### 3.2 Overall results

A total of 4,174 qualitative productions were produced by 1,068 children, of which 47% were girls and 53% were boys, all aged between 6 and 18 years. Qualitative analysis of the children's and teenagers' self-expression and storytelling through images revealed that they mentioned 30 determinants of health and cancer. The three most frequently mentioned determinants were “Consumption of psychotropic drugs”, “Diet”, and “Harmful environment”.

Among these 30 determinants of health and cancer identified by children and adolescents, there were significant gender differences in the way they perceived the determinants of health and cancer for 14 determinants: “Protective environment”, “Psychological wellbeing”, “Diet”, “Unhealthy diet”, “Screen/Audiovisual pollution”, “Curative”, “Preventive”, “Misuses”, “Self-image”, “Prevention messages”, “Animals (favorable)”, “Animals (unfavorable)”, “Children”, and “Vulnerability”. All these determinants were mentioned significantly in favor of girls.

Sub-group analyses [elementary school group (ages 6–11 years), middle school group (ages 11–16 years), and high school group (ages 15–18 years)] showed that the differences in conceptions between girls and boys evolved with age:

- In elementary schools (ages 6–11 years), girls gave significantly more importance (*p* < 0.05) to 11 determinants, while boys attached significantly more importance (*p* < 0.05) to two determinants: “Physical activities and sports” and “Leisure activities”.
- In middle school (ages 11–16 years), girls attached significantly more importance (*p* < 0.05) to 12 determinants, while boys gave significantly (*p* < 0.05) more importance to 1 determinant, “Physical activities and sports”.
- In secondary schools (aged 15–18 years), girls gave significantly (*p* < 0.05) more importance to 13 determinants. There was no significant difference (*p* < 0.05) in favor of boys for high school students.

### 3.3 Trends in the number of health and cancer determinants cited by girls and boys in the ages of 6–18 years

**Figure 2:**
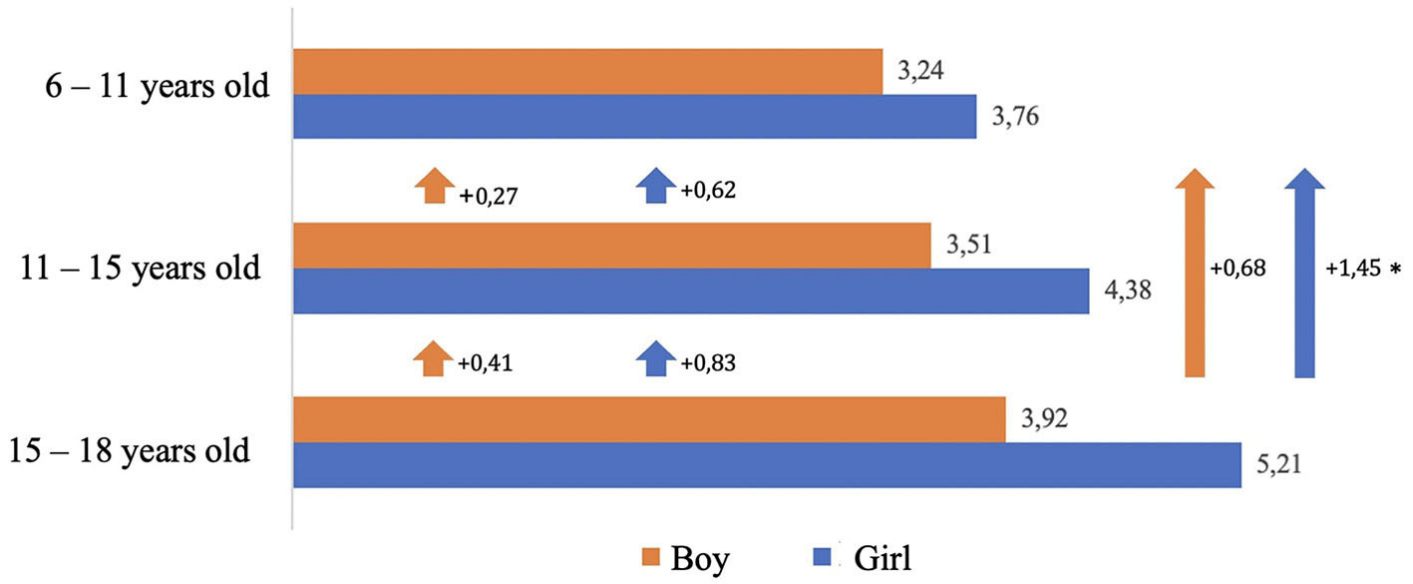
Changes in the number of health and cancer determinants cited by girls and boys as they advance in age (6–18) *p < 0.005.

The results of phases 1 and 2 reveal that girls mention an average of 4.45 determinants of health and cancer and boys mention 3.56 determinants (Figure 2).

Girls have a more systemic view of health determinants than boys. Intra-group analysis shows that the number of determinants cited by girls and boys increases with age. The increase in the number of determinants cited by girls is significant (*p* = 0.017) between the ages of 6–11 and 15–18 years. This means that girls' visions become richer than boys' as they get older.

This gap widens with age in favor of girls:

- From ages 6 to 15 years: (+ 0.62 determinants) for girls and (+ 0.27 determinants) boys.
- 11 to 18 years: (+ 0.83 determinants) for girls and (+ 0.41 determinants) boys.
- 6 to 18 years: (+ 1.45 determinants) for girls and (+ 0.68 determinants) boys.

Girls have a more holistic approach to health than boys from the age of 6 years. This phenomenon increases with age. Intergroup analysis shows that the gap between the number of determinants cited by girls and boys increases between the ages of 6 and 18 years.

- Girls in the ages of 6–11 years cite 0.52 more determinants than boys.
- Girls in the ages of 11–15 years cite 0.87 more determinants than boys.
- Girls in the ages of 15–18 years cite 1.29 more determinants than boys.

### 3.4 Pictorial representation of significant differences in conceptions of health determinants and cancers between girls and boys in the ages of 6–11 years

**Figure 3:**
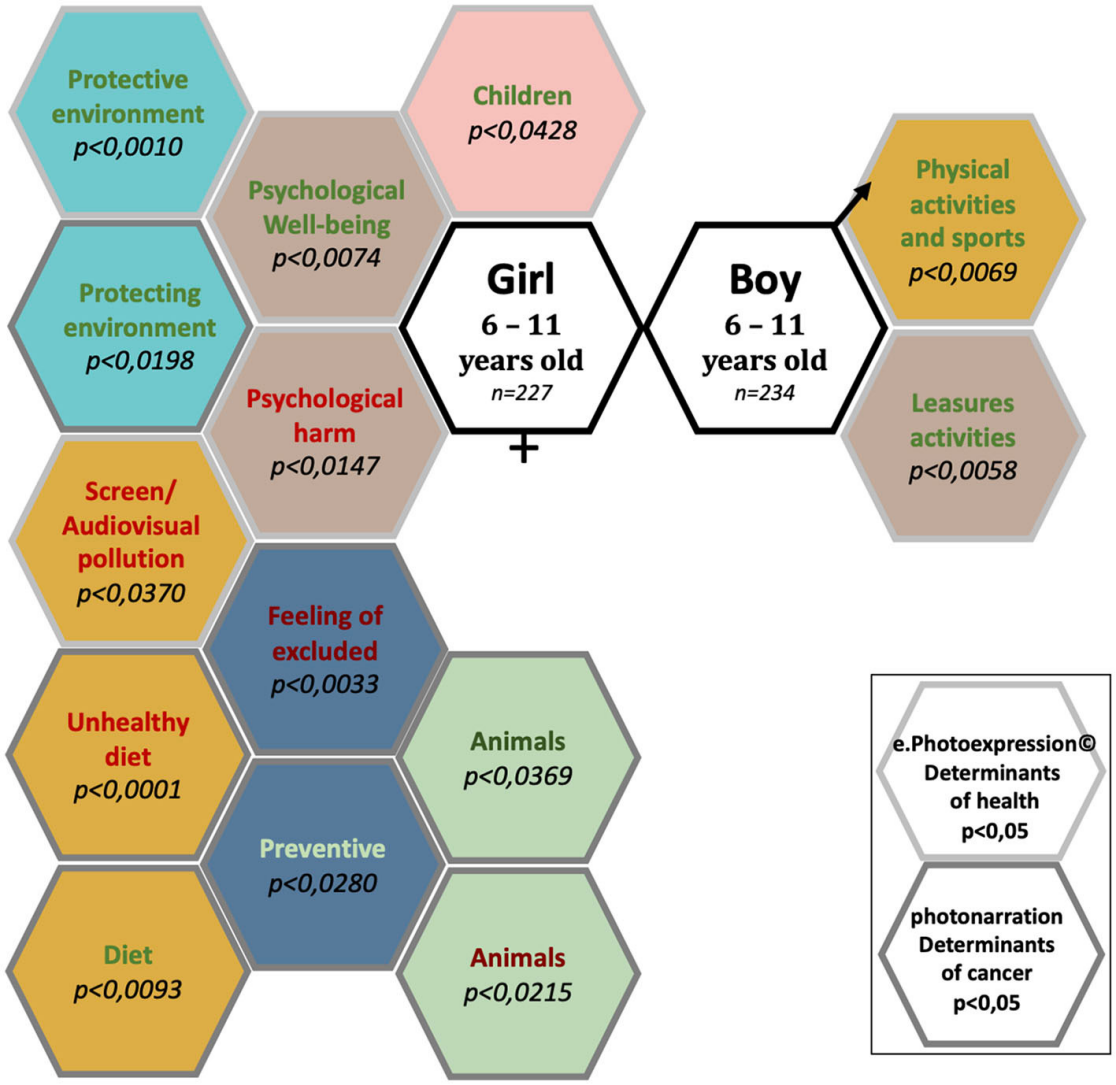
Conceptions of the determinants of health and cancer: significant differences between girls and boys aged 6–11 years, enrolled in elementary schools.

A total of 461 children in the ages of 6–11 years identified 28 determinants of health and cancer (Figure 3). Of these, there were significant gender differences in conceptions of the determinants of health and cancer for 13 determinants.

The determinants of health and cancer mentioned significantly (*p* < 0.05) by girls in the ages of 6–11 years were “Children”, “Diet”, “Unhealthy food”, “Screen/audio-visual pollution”, “Preventive”, “Feeling of exclusion”, “Psychological wellbeing”, “Psychological harm”, “Protective environment”, “Protecting the environment”, “Animals (favorable)”, and “Animals (unfavorable)” (12 determinants).

The determinants of health and cancer mentioned significantly (*p* < 0.05) by boys in the ages of 6–11 years were “Physical and Sports Activities” and “Leisure Activities” (2 determinants).

The determinants of health and cancer mentioned with no significant difference between girls and boys in the ages of 6–11 years were “Harmfull environment”, “Airing out”, “Sedentary”, “Physical activity and sports (unfavorable)”, “Curative”, “Misuses”, “Epidemic”, “Prevention message (favorable)”, “Prevention message (unfavorable)”, “Feeling of forbidden”, “Social link/family”, “To be able to do”, “Consumption of Psychotropic drugs”, and “Death” and “Vulnerability” (15 determinants).

### 3.5 Pictorial representation of significant differences in conceptions of health determinants and cancers between girls and boys in the ages of 11–15 years

**Figure 4:**
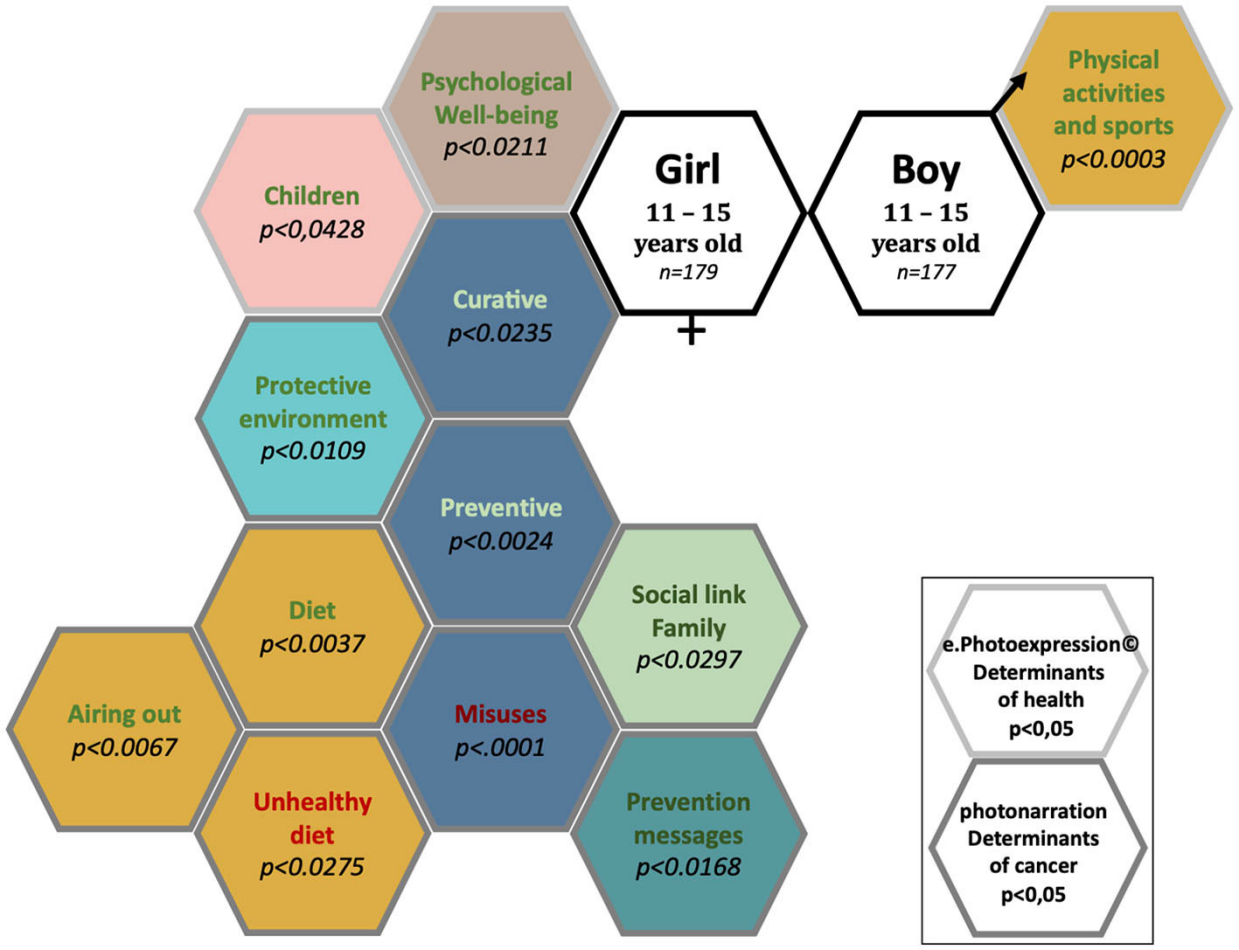
Conceptions of the determinants of health and cancer: significant differences between girls and boys aged 11–15 years attending secondary school.

A total of 356 children and adolescents in the ages of 11–15 years identified 30 determinants of health and cancer (Figure 4). Of these, there were significant differences in conceptions for 13 determinants.

The determinants of health and cancer mentioned significantly (*p* < 0.05) by girls in the ages of 11–15 years were “Children”, “Diet”, “Psychological wellbeing”, “Protective environment”, “Unhealthy diet”, “Curative”, “Preventive”, “Social link/family”, “Prevention message (favorable)”, “Airing out”, and “Misuses” (11 determinants).

The determinant of health and cancer mentioned significantly (*p* < 0.05) by boys aged 11 to 15 years was “Physical activities and sports” (1 determinant).

The determinants of health and cancer mentioned with no significant difference between girls and boys in the ages of 11–15 years were “Protecting the environment”, “Harmful environment”, “Leisure activities”, “Psychological harm”, “Screen/audio-visual pollution”, “Epidemic”, “Prevention message (unfavorable)”, “Feeling of forbidden”, “Animals (favorable)”, “Animals (unfavorable)”, “To be able to do”, “Consumption of psychotropic drugs”, “Death”, “Vulnerability”, “Feeling excluded”, “Sedentary”, and “Physical activity and sports (unfavorable)” (17 categories).

### 3.6 Pictorial representation of significant differences in conceptions of health determinants and cancers between girls and boys in the ages of 15–18 years

**Figure 5:**
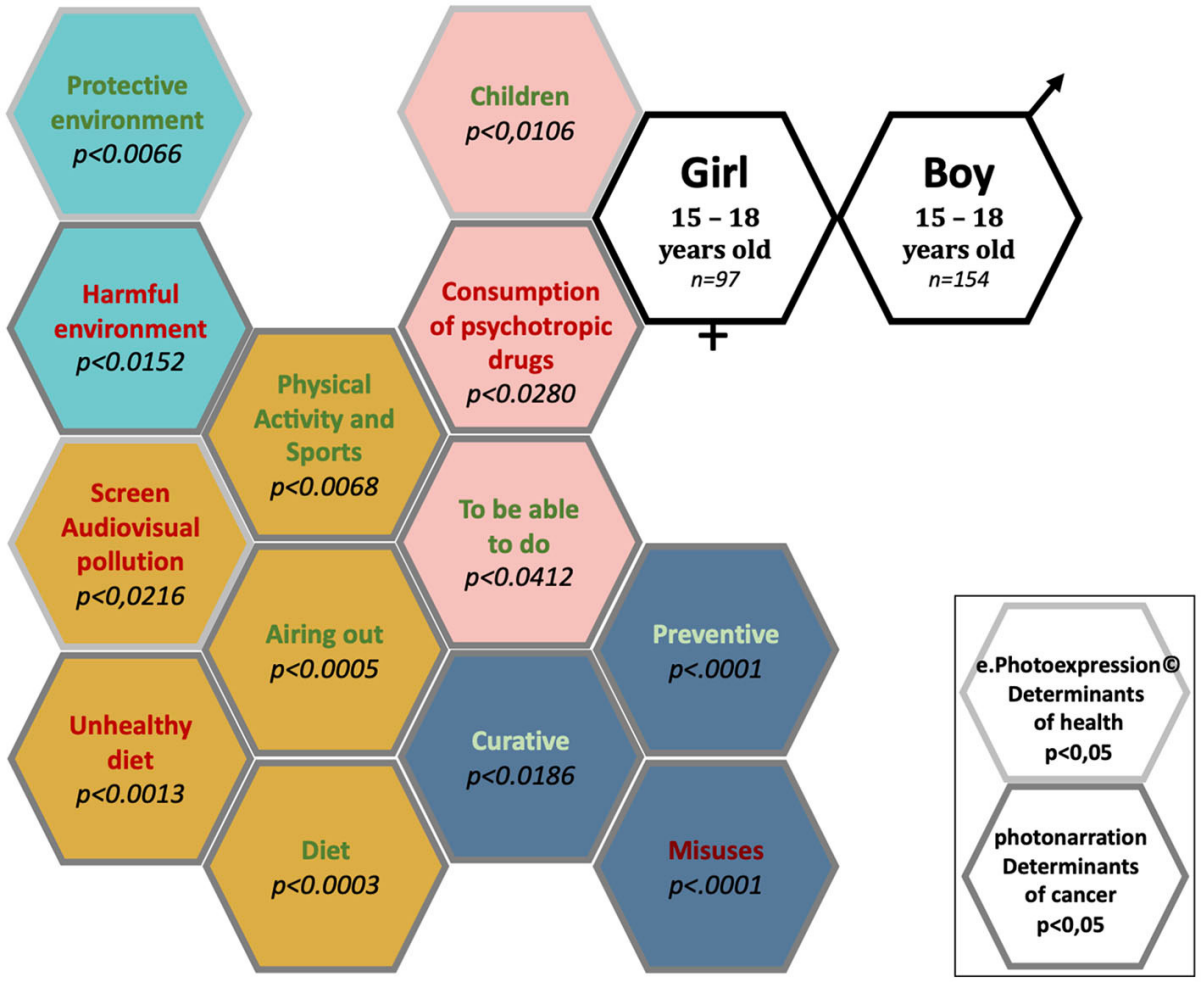
Conceptions of the determinants of health and cancer: significant differences between girls and boys aged 15–18 years attending secondary school.

A total of 251 children and adolescents identified 30 determinants of health and cancer (Figure 5). Of these, there were significant gender differences in conceptions of the determinants of health and cancer for 13 determinants.

The determinants of health and cancer mentioned significantly (*p* < 0.05) by girls in the ages of 15–18 years were “Children”, “Diet”, “Unhealthy diet”, “Screen/audio-visual pollution”, “Physical activity and sports”, “Consumption of psychotropic drugs”, “To be able to do”, “Preventive”, “Curative”, “Misuses”, “Protective environment”, “Harmful environment”, and “Airing out” (13 determinants).

No determinants of health or cancer were mentioned significantly (*p* < 0.05) by boys in the ages of 15–18 years.

The determinants of health and cancer mentioned with no significant difference between girls and boys in the ages of 11–15 years were “Protecting the environment”, “Leisure activities”, “Psychological wellbeing”, “Psychological harms”, “Physical activity and sports (deleterious)”, “Sedentary”, “Epidemic”, “Prevention messages (favorable)”, “Prevention messages (unfavorable)”, “Feeling of forbidden”, “Social link/family”, “Animals (favorable)”, “Animals (unfavorable)”, “Feeling excluded”, “Death”, and “Vulnerability” (16 determinants).

### 3.7 Pictorial representation of the evolution of significant differences in conceptions of health determinants and cancers between girls and boys in the ages of 6–18 years

**Figure 6:**
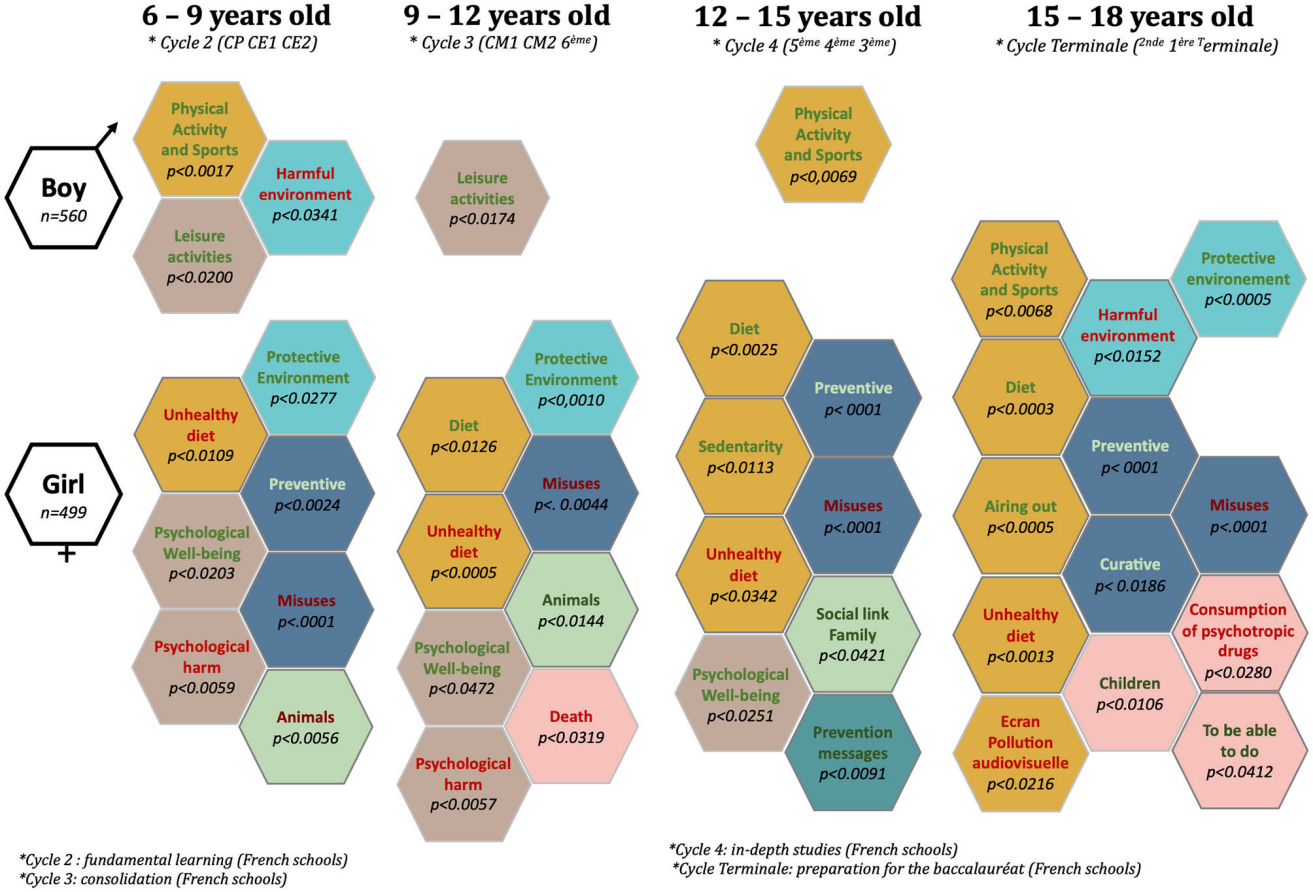
Evolution of conceptions of the determinants of health and cancer with advancing age: significant differences between girls and boys aged 6–18 years.

The significant differences between girls' and boys' understanding of the determinants of health and cancer evolve and intensify with advancing age (Figure 6).

Between the ages of 6 and 18 years, the number of determinants significantly mentioned by boys decreased (from 3 to 0). At the age of 9, the decline was most pronounced: from 3 to 1 determinant.

Conversely, the number of determinants significantly mentioned by girls increased over the course of their schooling (from 7 to 13 determinants). At the age of 15, the increase was most pronounced: from 8 to 13 determinant.

The determinants “Misuses” and “Unhealthy diet” were mentioned significantly by girls aged 6 to 18 years.

Girls also attached importance to “Psychological wellbeing” (ages 6–15 years), “Protective environment” (ages 6–12 and 15–18 years), “Diet” (ages 9–18 years), and “Prevention” (ages 6–9 and 12–18 years).

The determinant “Psychological harm” was mentioned significantly by girls in the ages of 6–12 years.

The determinant “Animals (unfavorable)” was mentioned significantly by girls in the ages of 6–9 years.

The determinants “Animals (favorable)” and “Death” were mentioned significantly by girls in the ages of 6–12 years.

The determinants “Social ties”, “Prevention messages”, and “Sedentary” were mentioned significantly by girls in the ages of 12–15 years.

The determinants “Children”, “To be able to do”, “Consumption of psychotropic drugs”, “Physical activity and sports”, “Airing out”, “Harmful environment”, and “Curative” were mentioned significantly by girls in the ages of 15–18 years.

## 4 Discussion

### 4.1 Main results

The results of this research are transferable to prevention intervention. They provide elements for understanding the conceptions that children and adolescents mobilize for their health. The e.Photoexpression© is proving to be a decision-making tool in the field of health, enabling us to implement strategies with children and adolescents based on the conceptions and representations they express. Mapping health conceptions enables us to propose prevention priorities that are adapted and targeted to the group's needs.

This study provides currently non-existent data on what children and adolescents think about health. The large number of children interviewed qualitatively is also innovative. An analysis of the 4,174 self-expression and image-based narrative productions produced by 1,068 children revealed significant differences in conceptions of the determinants of health and cancer according to gender and age.

Girls have a more systemic view of health determinants than boys. This phenomenon increases with age. From an early age, girls take a more holistic approach to health than boys. The increase in the number of determinants cited by girls was significant (*p* = 0.017) between the ages of 6–11 and 15–18 years.

Thus, the vision of what determines good and bad health expands more significantly for girls than for boys as they get older.

The number of determinants significantly mentioned by girls increased over the course of their schooling (from 7 to 13 determinants). This increase was most pronounced at the age of 15 years: from 8 to 13 determinants. Conversely, the number of determinants mentioned by boys significantly decreased between ages 6 and 18 years (from 3 to 0 determinants). This reduction was most pronounced at the age of 9 years: from 3 to 1 determinants.

The determinants mentioned significantly by boys were physical activity (ages 6–9 and 12–15 years) and leisure (ages 6–12 years). Girls attached more importance to healthy (ages 9–18 years) or unhealthy (ages 6–18 years) food, psycho-emotional wellbeing (ages 6–15 years), a protective environment (ages 6–12 and 15–18 years), misuse of healthcare (ages 6–18 years), and the importance of a healthy lifestyle as a preventive factor (ages 6–9 and 12–18 years).

Overall, in the ages of 6–18 years, girls were significantly more focused on 19 determinants, while boys are more focused on 3 determinants.

This study has its strengths. It is a qualitative study involving a very large number of children and adolescents. The collection and analysis of data generated a mass of qualitative data, which is unprecedented and innovative. The high density of data collected and analyzed provides new, unprecedented, and crucial information for understanding children's and teenagers' conceptions of the determinants of health and cancer. This study provides the first mapping of the differences between girls' and boys' perceptions of health and cancer. It also provides a basis for explaining pathways (14). If we are to “grow up well” and “age well”, we need to take account of these differences in perception from childhood onward in our prevention policies.

The main limitation of this study is that it was carried out in a single French region, the Auvergne-Rhône-Alpes. It would be interesting to conduct it on a national and international scale to compare the conceptions of children from different countries. Other contextual elements were measured, such as the geographical location of schools and the social and economic parameters of families. The presentation of these data will be the subject of a future publication.

Qualitative studies looking at children's conceptions of health and illness show that young and older children have a holistic view of health (15–17).

Other studies examine children's perceptions of health but focus on specific themes such as sugar (18), physical activity (13, 19), and mental health (13).

As previously stated, there are very few qualitative studies of children's and adolescents' conceptions of the determinants of health. One study explores differences between the views of adolescents and adults (20), but gender differences have not been explored. One quantitative study used the Child-SOC to measure children's perceived health (15). It explored the differences between girls and boys. The results indicate some differences between girls and boys in the ages of 8–10 years. This difference was not significant but indicates that boys have a higher sense of coherence associated with perceived health than girls.

The following paragraphs will shed light on gender differences according to documented themes on this subject and in relation to the results of this study: health behaviors, mental health, physical activity, and leisure.

Gender has an important influence on health knowledge and behavior (21). Data exist on the health behaviors of the adult population. It appears that women are more attentive to their health than men and that their behaviors are more favorable to their health (22, 23). In fact, they are more informed, more interested in prevention, and consult their GP more regularly (22). Despite having a higher life expectancy at birth than men (85.2 vs. 79.2 years) and a slightly higher disability-free life expectancy (DFLE) than men (67 vs. 65.6 years), they declare themselves to be in poorer health than men (22) and live longer with disabilities. On average, women spend 21 years with moderate or severe disabilities and men spend 16 years (24). In short, “women now make up the majority of people in precarious situations”.

Our study shows that girls in the ages of 6–18 years attach greater importance to many health determinants than boys. Thus, gender differences in health perceptions are present from a very early age and can help to explain life paths. For “growing up well” and “aging well”, prevention policies need to take account of these differences in perception from childhood onwards. This is the first study to examine children's and teenagers' conceptions of their overall health and to identify gender-related differences in perceptions. The influence of gender on schooling has been extensively studied (25). Girls in elementary school are described as being more interested and invested in learning than boys. Girls' confidence in their abilities and motivation decline over the course of school. Between third and sixth grades, social elements deteriorate more for girls than boys (26). Boys have a higher perception of general self-esteem and skills (academic, athletic, and physical) than girls (27). Girls are more likely to opt for careers in health-related fields, while boys go for other scientific fields (28). These phenomena can be explained by various theories. The reproduction of social inequalities can explain this paradox across the world through the role of the school system, family, and society (28, 29). Career paths, as the result of individual strategies, can also explain this phenomenon (30). By negatively anticipating their chances of success, girls may resort to self-censorship. Boys' lesser appetite for paramedical and social fields is explained by the lack of visibility of men who have chosen these fields (33%), the apprehension of an essentially female environment (32%), and the influence of their entourage (parents and family) (31%) (31).

These theories shed light on the results of our study. For example, girls' more systemic view of health determinants than boys' may be explained by their greater investment in learning and appetence for health-related subjects in contrast to boys.

A study of the mental health perceptions of young people between the ages of 16 and 19 years shows that girls experience the negative aspects of social processes, social interactions, performance, and responsibility. Boys' more positive mental health is associated with their lower levels of responsibility. Cultural norms of femininity and masculinity are also more complex for girls. These differences put them at greater risk of mental health problems (32).

Girls are more likely to express concern about being perceived as weak or different, but compared with boys, they feel less likely to have their identity challenged (33).

These social factors and processes, gender relations, and constructions of masculinity and femininity should be recognized as important for adolescent mental health.

Physical activity and the fight against sedentary lifestyles are public health issues (34, 35). Our study shows an overall focus on health determinants for girls and an interest in physical activity and leisure for boys. WHO recommendations correspond to an accumulation of at least 60 min of moderate-to-vigorous physical activity per day (36). Among children and adolescents aged 6–17 years, 50.7% of boys and 33.3% of girls meet these recommendations (37, 38). Physical activity declines with advancing age, and even more so during adolescence, with 16% of girls and 40% of boys aged 15–17 years. Gender is a major determinant of physical activity. The decline in physical activity is even more marked among girls (29) and can be explained by the influence of social norms, body transformation, the female image, and the influence of peers and family (39). This (40) social shaping is present from the earliest age, from 6 years onwards (41). Girls' vision of physical activity is hygienic, with a preference for health-related activities and self-maintenance (42). This literature is consistent with the results of our study.

Our study shows that between the ages of 6 and 12 years, boys attach greater importance to leisure activities than girls. These data echo the study by Maruejouls, who highlights the unequal sharing of leisure spaces in favor of boys, starting in the playground (43). An SRVC survey (44) shows that men are more satisfied with leisure activities (2010–2019) than women. Over the past 10 years, this gap has been narrowing. Between the ages of 20 and 35 years, which is the relative age at which children arrive in the home, the difference in satisfaction between women and men in the area of leisure is greatest (44). Our study shows that, from an early age, boys have a favorable perception of leisure activities. These perceptions will influence life trajectories and the place given to leisure activities by women and men at different stages of their lives. Taking these perceptions into account from an early age to limit societal influence and the standardization of discourse is vital in view of the importance of these activities for health and wellbeing.

### 4.2 Conclusion

This study shows the existing differences in conceptions linked to gender and age. These differences evolve with advancing age and are not fixed. This research provides data on how children do or do not enter into systemic thinking about what positively or negatively influences their health status and the occurrence of cancer. Girls have a more global vision of the determinants of health, and this gap in discourse widens as they get older.

Boys focus on physical and leisure activities, while girls take a more holistic view, attaching greater importance to diet (healthy or unhealthy), psycho-emotional wellbeing, protective environment, care, and hygiene. We demonstrate that societal actions influence and normalize the vision of girls and boys from the earliest age: 6 years.

To limit these impacts, it seems essential to think differently about prevention.

This research describes how to identify a set of indicators to help in healthcare decision-making. Indeed, we demonstrate that a population diagnosis before any prevention intervention is essential to prioritize, target, and adapt interventions to a heterogeneous public and thus to differentiate interventions when the public's conceptions diverge and eventually to delve deeper into subjects that bring them closer together. The degree of importance of the comments collected and their disparities enable us to identify priority areas for action. Indeed, to be effective with the greatest number of people, it is preferable to avoid pre-established interventions on a precise theme, without taking into account conceptions that are too far removed from the specificities of individuals. Gathering conceptions enables us to act as close as possible to the zone of proximal development (7, 45) and thus to mark out anchor points that are close to children's conceptions. Consideration of the individual's own conceptions reinforces empowerment and thus promotes health-promoting decision-making. In fact, if the theme is not linked to the public's knowledge, the intervention will be less effective. Analyzing participants' profiles saves valuable time in identifying the group's needs. The prevention coordinator can identify trends, such as the most fragile individual profiles, those who develop the most, and the overall profile of the group, and thus act as closely as possible to the participants' conceptions.

When it comes to prevention, it is important to take gender, development, and age factors into account. These disparities have a methodological impact on intervention design. This information makes it possible to individualize and adapt the session to any audience. The facilitator can draw on the strongest profiles to help the most fragile. A gendered approach based on age, investigating prevention themes that are less attractive to boys with the help of girls and vice versa while remaining nuanced in the discourse, is an example of an intervention that would have an effective impact. Rapidly identifying the perception of favorable and unfavorable determinants of health will make it possible to focus on more than just favorable determinants. Indeed, the negative should not be taboo, and it is important to verbalize it in prevention, even if it occupies a predominant place in the participants' conceptions.

Consequently, this research data, which can be transferred to current interventions and practices, is a powerful decision-making tool for prevention workers, enabling them to act effectively, as close as possible to the conceptions and needs of participants. This contribution to knowledge justifies the need to constantly adapt prevention interventions to the challenges publicly encountered.

## Data availability statement

The original contributions presented in the study are included in the article, further inquiries can be directed to the corresponding author.

## Author contributions

CG: Writing – original draft. MD: Writing – review & editing. PB: Software, Writing – review & editing. LG: Visualization, Writing – review & editing. FP: Conceptualization, Supervision, Writing – review & editing.
